# Grammar-based compression approach to extraction of common rules among multiple trees of glycans and RNAs

**DOI:** 10.1186/s12859-015-0558-4

**Published:** 2015-04-24

**Authors:** Yang Zhao, Morihiro Hayashida, Yue Cao, Jaewook Hwang, Tatsuya Akutsu

**Affiliations:** Bioinformatics Center, Institute for Chemical Research, Kyoto University, Gokasho, Uji Japan

**Keywords:** Grammar-based compression, Bisection-type tree grammar, Glycan, RNA secondary structure

## Abstract

**Background:**

Many tree structures are found in nature and organisms. Such trees are believed to be constructed on the basis of certain rules. We have previously developed grammar-based compression methods for ordered and unordered single trees, based on bisection-type tree grammars. Here, these methods find construction rules for one single tree. On the other hand, specified construction rules can be utilized to generate multiple similar trees.

**Results:**

Therefore, in this paper, we develop novel methods to discover common rules for the construction of multiple distinct trees, by improving and extending the previous methods using integer programming. We apply our proposed methods to several sets of glycans and RNA secondary structures, which play important roles in cellular systems, and can be regarded as tree structures. The results suggest that our method can be successfully applied to determining the minimum grammar and several common rules among glycans and RNAs.

**Conclusions:**

We propose integer programming-based methods MinSEOTGMul and MinSEUTGMul for the determination of the minimum grammars constructing multiple ordered and unordered trees, respectively. The proposed methods can provide clues for the determination of hierarchical structures contained in tree-structured biological data, beyond the extraction of frequent patterns.

**Electronic supplementary material:**

The online version of this article (doi:10.1186/s12859-015-0558-4) contains supplementary material, which is available to authorized users.

## Background

Many tree structures are found in nature and organisms. One such tree structure is a glycan, in which each monosaccharide is regarded as a vertex, except for cyclic oligosaccharides and so on. Since glycans contain various complicated structures, they are believed to be constructed by various mechanisms that recognize a monosaccharide, binding it with another. For instance, a galactosyltransferase is known to catalyze the biosynthesis with a galactose [1]. Glycans are also known to play several important roles in a cellular system, such as molecular recognition, cell adhesion, and antigen-antibody interactions. Therefore, many studies have been conducted to understand the structures and functions of glycans; in addition, several methods have been developed for the discovery of glycan motifs or significant subtrees, as glycan structures are conserved in evolutionary processes [2-4], and to measure the similarities between glycans [5,6].

RNA secondary structures can be also regarded as tree structures; these structures depend on the nucleic acid sequence. RNAs, which are large biological molecules, also perform important functions in living cells, such as the catalysis of biological reactions and expression of genes. Milo et al. displayed a pseudoknot-free RNA secondary structure as an ordered rooted tree, wherein each base pair, unpaired base interval, hairpin loop, internal loop, multi-loop, and external loop corresponds to a vertex, and developed a cubic time algorithm for the homeomorphic subtree alignment problem [7]. They applied it to pairwise alignments of RNA secondary structures, and found several structural similarities, which were not detected by other existing algorithms. Chen and Zhang developed an efficient algorithm for tree edit distance, and used the same to compare several RNA secondary structures [8]. These methods tried to measure the similarities between the tree structures, and to determine frequent subtrees.

**Figure 1:**
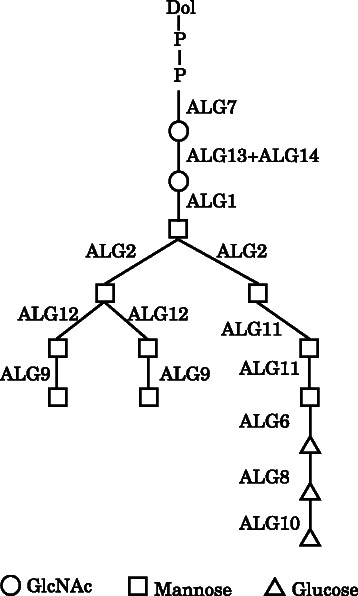
Glycosyltransferases involved in the linkages of the Glc _3_ *Man*_9_ *GlcNAc*_2_ oligosaccharide precursor [9]. GlcNAc denotes N-acetylglucosamine.

In this paper, we focus on finding construction rules for multiple biomolecular tree structures. For example, it was reported that the glycosyltransferases such as ALG1 and ALG2 are involved in the linkages of Glc _3_ *Man*_9_ *GlcNAc*_2_ oligosaccharide precursor as shown in Figure 1 [9], where Glc, Man, and GlcNAc stand for glucose, mannose, and N-acetylglucosamine, respectively. ALG1 connects a GlcNAc with a Man, and ALG2 connects a Man with the Man connected by ALG1. According to local structures, different glycosyltransferases catalyze those biosyntheses in order to construct the same structure of the oligosaccharide. Since it is difficult to find existences of such genes from one tree structure, we try to find them from multiple tree structures that the same enzyme constructs a specified local structure.

In the field of computer science, grammar-based compression is used to determine the rules of construction of various types of data. The identification of the smallest grammar for input data would provide a clue towards understanding the construction rules. The determination of the smallest Context-Free Grammar (CFG) constructing a given string is known to be NP-hard [10]. Polynomial-time approximation methods have been developed to determine the smallest CFG for sequence data such as DNA, RNA nucleic acid sequences, and protein amino acid sequences [10-13]. Although these methods were extended to the tree structured data including XML [14-16], the minimum grammar has not always been provided. Hence, in our previous study, we developed grammar-based compression methods for single trees that always output the minimum grammar. We used bisection-type tree grammars proposed in [17], Simple Elementary Ordered Tree Grammar (SEOTG) for ordered trees and Simple Elementary Unordered Tree Grammar (SEUTG) for unordered trees, in which at most two nonterminal and terminal symbols appear on the right-hand side of each production rule. Since it seemed to be difficult to directly formulate the problems of finding the minimum SEOTG and SEUTG using Integer Programming (IP), we instead formulated the problems of finding SEOTG and SEUTG with a given size [18].

We previously developed methods for compressing single trees. As considered in the example provided in Figure 1, specified construction rules can be applied to generate several similar trees. Therefore, in this study, we attempt to discover common construction rules among multiple distinct trees; in addition, by improving and extending the previous methods, we propose the novel IP formulations MinSEOTGMul and MinSEUTGMul, for the direct determination of the minimum grammars of SEOTG and SEUTG. In our previous study, the problems associated with the determination of the minimum SEOTG and SEUTG were not directly formulated by IP; instead, the problems associated with determining the SEOTG and SEUTG utilizing the given sizes were formulated. Therefore, the previous IP was executed at least twice with different parameters in order to confirm the minimum size of the grammars that construct a given single tree. The methods proposed in this paper can be applied to the direct determination of the minimum grammar in one attempt. As for multiple input trees, our previous method can be trivially extended for the determination of the minimum SEOTG and SEUTG for *N* trees, which adds a special root vertex, connects each root of *N* trees to the special root by a distinct special edge, and applies the previous IP formulation to this generated single tree. This approach, however, increases the number of variables in the IP formulation, and may enlarge the execution time.

We apply our methods to several sets of glycans and RNA secondary structures. Consequently, we successfully determined the minimum grammar and several common construction rules using this method.

## Methods

In this section, we briefly review the CFG, SEOTG, and SEUTG [17], and explain the proposed IP formulations, MinSEOTGMul and MinSEUTGMul, for multiple trees. Integer Programming (IP) is a method used to optimize a linear objective function subject to linear inequality constraints, with the variables being restricted to integers. We use these tools to solve the proposed integer program as our problem of finding the minimum SEOTG and SEUTG is NP-hard, with efficient solvers being developed. The benefit to use IP is that we can obtain exact solutions for combinatorial optimization problems.

### Context-free grammar (CFG)

A context-free grammar (CFG) deals with strings, and is represented by 4-tuple (*T*,*V*,*S*,*P*), where *T* is a set of terminal symbols, *V* is a set of nonterminal symbols, *S* is a start symbol in *V*, and *P* is a set of production rules, wherein a nonterminal symbol on the left-hand side is replaced with a string on the right-hand side, which consists of symbols from *V* and *T* [19]. The final product generated by a CFG does not include any nonterminal symbol. The size of a grammar is defined as the total number of symbols appearing on the right-hand side of the production rules. For example, in a case where *T*={*a*,*b*}, *V*={*S*}, and *P*={*S*→*a* *S* *b*,*S*→*a* *b*}, the start symbol *S* is repeatedly replaced with the rule *S*→*a* *S* *b*, all nonterminal symbols are replaced with terminal symbols, and the strings ’ab’, ’aabb’, ’aaabbb’, etc. are generated, by this grammar. The size of the grammar is 5. In a case where *T*={*a*,*b*}, *V*={*S*,*X*,*Y*}, and *P*={*S*→*a* *X* *b*,*X*→*a* *Y* *b*,*Y*→*a* *b*}, only ’aaabbb’ is generated from *S*. The size of this grammar is 8. *X* and *Y* represent ’aabb’ and ’ab’, respectively. The rest of this paper deals with grammars that generate a constant number of trees. Each nonterminal symbol represents a specified tree.

### Simple elementary ordered/unordered tree grammar (SEOTG/SEUTG)

**Figure 2:**
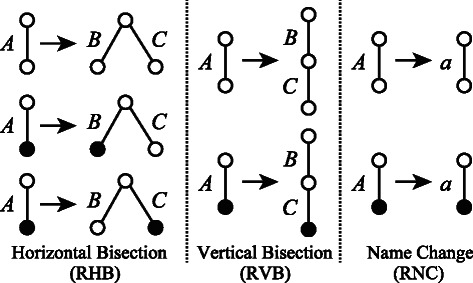
Production rules for Simple Elementary Ordered Tree Grammar (SEOTG). (RHB) Horizontal bisection rules, (RVB) Vertical bisection rules, (RNC) Replacement rules with a terminal symbol. The uppercase letters, ’A’, ’B’, and ’C’ indicate non-terminal symbols, while the lowercase letter, ’a’, indicates a terminal symbol. The circle filled in black indicates a tag.

**Figure 3:**
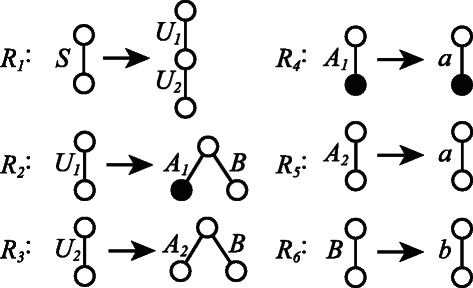
Example of SEOTG with (*Σ*,*Γ*,*S*,*Δ*). *Σ* indicates a set of terminal symbols, *Γ* indicates a set of nonterminal symbols *S*,*U*_1_,*U*_2_,*A*_1_,*A*_2_,*B*, and *Δ* indicates the set of six production rules, *R*_1_,⋯,*R*_6_.

**Figure 4:**
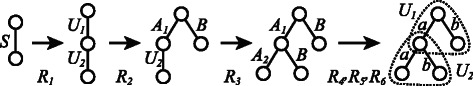
Derivation of tree *T* using the grammar, with the set of production rules shown in Figure 3. The trees surrounded by the dotted curves are derived from nonterminal symbols *U*_1_ and *U*_2_, respectively.

Simple elementary ordered tree grammar (SEOTG) is defined for the rooted ordered trees *T*(*V*,*E*), where *V* is a set of vertices, and *E* is a set of labeled edges. If *T* represents a glycan, a vertex corresponds to a monosaccharide, an edge corresponds to a bond between the monosaccharides, and the enzyme involved in the biosynthesis can be represented by the label of the edge, as shown in Figure 1. As well as CFGs, a grammar of SEOTG consists of 4-tuple (*Σ*,*Γ*,*S*,*Δ*), where *Σ* is a set of terminal symbols, *Γ* is a set of nonterminal symbols, *S* is a start symbol in *Γ*, and *Δ* is a set of production rules, that are classified into Horizontal Bisection (RHB), Vertical Bisection (RVB), and Name Change (RNC), as in Figure 2. It should be noted that production rules of SEOTG and SEUTG are different from construction rules of biomolecular trees. (RHB) includes three rules that an edge of nonterminal symbol *A* is replaced with a tree whose root is both roots of nonterminal symbols *B* and *C*. *A* is bisected at the root into *B* and *C*. We introduce *a tag* to represent the vertex connected with another tree. The first rule in (RHB) does not contain a tag, and the other rules contain a tag, respectively. (RVB) includes two rules that an edge of nonterminal symbol *A* is replaced with a tree in which the root is the root of nonterminal symbol *B*, and the root of nonterminal symbol *C* is attached to the tag of *B*. *A* is bisected at an internal vertex of *A* into *B* and *C*. (RNC) includes two rules that an edge of nonterminal symbol *A* is replaced with an edge of terminal symbol *a*. In addition, any nonterminal symbol does not appear in expansion of the symbol itself. Then, each nonterminal symbol corresponds to a subtree of a given tree *T*. Figure 3 shows an example of SEOTG with (*Σ*,*Γ*,*S*,*Δ*). *Δ* is the set of six production rules, *R*_1_,⋯,*R*_6_, where *R*_1_ is a vertical bisection rule, *R*_2_ and *R*_3_ are horizontal bisection rules, and *R*_4_,*R*_5_,*R*_6_ are name change rules. Figure 4 illustrates the derivation of a tree from the start symbol *S* by the SEOTG. The first replacement is done by *R*_1_, and *U*_1_ is replaced with the right-hand side of *R*_2_. Then, the lower endpoint of *U*_1_ connects with the root of *U*_2_, and one of leaves of the replaced tree of *U*_1_ connects with the root of *U*_2_. A tag indicates the vertex connected with another vertex. Hence, the lower endpoint labeled with a tag in *A*_1_ connects with the root of *U*_2_. By applying *R*_3_,⋯,*R*_6_, the right-most tree is generated. The trees surrounded by dotted curves are derived from nonterminal symbols *U*_1_ and *U*_2_, respectively. It is considered in the example of Figure 1 that a terminal symbol corresponds to a biosynthesis, and a nonterminal symbol corresponds to a sequence of biosyntheses.

Simple elementary unordered tree grammar (SEUTG) is defined for rooted unordered trees in a manner similar to the SEOTG. In SEUTG, the second and third production rules with a tag in the (RVB), described in Figure 2, are equivalent to each other, as the trees are unordered.

### Extension to multiple trees

**Figure 5:**
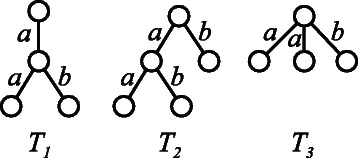
Example of input multiple trees *T*_1_, *T*_2_, and *T*_3_.

**Figure 6:**
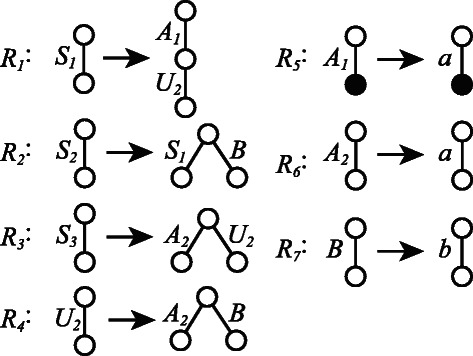
Minimum SEOTG with (*Σ*,*Γ*,{*S*_1_,*S*_2_,*S*_3_},*Δ*) that generates *T*_1_, *T*_2_, and *T*_3_, as shown in Figure 5. *S*_1_, *S*_2_, *S*_3_ are start symbols in this grammar, *Γ*={*S*_1_,*S*_2_,*S*_3_,*U*_2_,*A*_1_,*A*_2_,*B*}, and *Δ* is the set of seven production rules, *R*_1_,⋯,*R*_7_.

**Figure 7:**
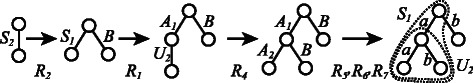
Derivation of the tree *T*_2_ shown in Figure 5 by the grammar, using the set of production rules shown in Figure 6. The trees surrounded by dotted curves are derived from nonterminal symbols *S*_1_ and *U*_2_, respectively.

We extend the SEOTG and SEUTG to multiple trees. *N* is the number of given trees, and *T*_*α*_ indicates the *α*-th edge labeled rooted tree. The start symbol *S* is replaced with the set of the nonterminal symbols *S*_*α*_. Each tree *T*_*α*_ is generated from *S*_*α*_ using one grammar. Figure 5 shows an example of the input multiple trees *T*_1_, *T*_2_, and *T*_3_. One of the minimum grammars generating these trees is shown in Figure 6. The size of the grammar is the total number of symbols present on the right-hand side in the production rules, i.e., 11. We minimize the number of distinct nonterminal symbols instead of the size of the grammar, as there exists the same number of production rules as the number of distinct nonterminal symbols. Figure 7 illustrates the derivation of *T*_2_ from the start symbol *S*_2_ using the grammar. *T*_2_ is the same as *T* in Figure 4, and we observe the modification of the derivation process by providing other similar trees.

The Euler string *e* *s*(*T*) is used to determine if the labeled rooted trees *T*_1_ and *T*_2_ are isomorphic to each other, where *e* *s*(*T*) for a tree *T* is defined by the sequence of edge labels *l* and its opposite $\bar {l}$, along the depth-first search traversal of *T* [17]. For example, for *T*_3_ in Figure 5, *e* *s*(*T*_3_) is determined to be $a\bar {a}a\bar {a}b\bar {b}$. For a tagged tree, the tagged edge, labeled *a*, is transformed into $a\tau \overline {a}$, using a special symbol, *τ*, which represents the tag. It is noted that for two edge labeled rooted trees *T*_1_ and *T*_2_, *T*_1_ is isomorphic to *T*_2_ if (and only if) *e* *s*(*T*_1_)=*e* *s*(*T*_2_).

*U* is assigned as the set of all Euler strings for all connected subtrees of *N* trees. By improving the previous formulation, we propose the following IP formulation, MinSEOTGMul, for the direct determination of the minimum SEOTG that constructs *N* ordered trees *T*_*α*_.

(1) $$\begin{array}{*{20}l} &\text{Minimize} \sum\limits_{u\in U}p_{u}\\ &\text{Subject to}\\ &x_{\alpha,i,\epsilon,j,j} = 1 \quad \text{for all } \alpha, i, j\in ch(\alpha,i) (|ch(\alpha,j)|=0),  \end{array} $$

(2) $$\begin{array}{*{20}l} &x_{\alpha,i,j,j,j}\, = 1 \quad \text{for all } \alpha, i, j\in ch(\alpha,i) (|ch(\alpha,j)|>0), \end{array} $$

(3) $$\begin{array}{*{20}l} &x_{\alpha,1,\epsilon,lch(\alpha,1),rch(\alpha,1)}=1 \text{ for all } \alpha, \end{array} $$

(4) $$\begin{array}{*{20}l} &x_{\alpha,i,\epsilon,h,k}\leq \sum_{l=h}^{k-1} y_{\alpha,i,\epsilon,h,l,k}+\sum_{t\in I(T_{\alpha,i,\epsilon,h,k})}z_{\alpha,i,\epsilon,h,k,t}\\ &\hspace*{54pt}\text{for all } \alpha, i, h \leq k \in ch(\alpha,i), \end{array} $$

(5) $$\begin{array}{*{20}l} &y_{\alpha,i,\epsilon,h,l,k}\leq \frac{1}{2}(x_{\alpha,i,\epsilon,h,l}+x_{\alpha,i,\epsilon,l+1,k}) \\ &\hspace*{54pt}\text{for all } \alpha, i, h \leq l<k \in ch(\alpha,i), \end{array} $$

(6) $$\begin{array}{*{20}l} &z_{\alpha,i,\epsilon,h,k,t}\leq \frac{1}{2}(x_{\alpha,i,t,h,k}+x_{\alpha,t,\epsilon,lch(\alpha,t),rch(\alpha,t)})\\ &\hspace*{54pt}\text{for all } \alpha, i, h \!\leq\! k \in ch(\alpha,i), t\!\in\! I(T_{\alpha,i,\epsilon,h,k}),  \end{array} $$

(7) $$\begin{array}{*{20}l} &x_{\alpha,i,j,h,k}\leq \sum_{l=h}^{k-1} y_{\alpha,i,j,h,l,k}+\sum_{t\in anc(\alpha, j)}z_{\alpha,i,j,h,k,t}\\ &\hspace*{54pt}\text{for all } \alpha, i, h\!\leq\! k\in ch(\alpha,i), j\in I(T_{\alpha,i,\epsilon,h,k}),  \end{array} $$

(8) $$\begin{array}{*{20}l} &y_{\alpha,i,j,h,l,k}\leq \frac{1}{2}(x_{\alpha,i,\epsilon,h,l}+x_{\alpha,i,j,l+1,k}) \\ &\hspace*{54pt}\text{for all } \alpha, i, h\leq l<k\in ch(\alpha,i),\\&\hspace*{6.9pc} j\in I(T_{\alpha,i,\epsilon,l+1,k}),  \end{array} $$

(9) $$\begin{array}{*{20}l} &y_{\alpha,i,j,h,l,k}\leq \frac{1}{2}(x_{\alpha,i,j,h,l}+x_{\alpha,i,\epsilon,l+1,k}) \\ &\hspace*{54pt} \text{for all } \alpha, i, h\leq l<k\in ch(\alpha,i),\\&\hspace*{6.8pc} j\in I(T_{\alpha,i,\epsilon,h,l}), \end{array} $$

(10) $$\begin{array}{*{20}l} &z_{\alpha,i,j,h,k,t}\leq \frac{1}{2}(x_{\alpha,i,t,h,k}+x_{\alpha,t,j,lch(\alpha,t),rch(\alpha,t)}) \\ &\hspace*{54pt} \text{for all } \alpha, i, h\!\leq\! k\in ch(\alpha,i), j\in I(T_{\alpha,i,\epsilon,h,k}), \\&\hspace*{6.8pc} t\in anc(\alpha, j), \end{array} $$

(11) $$\begin{array}{*{20}l} &s_{u} \leq p_{u}<1+s_{u}\quad \text{for all } u \in U, \end{array} $$

(12) $$\begin{array}{*{20}l} &s_{u}=\frac{1}{|\left\{(\alpha,i,j,h,k)|es(T_{\alpha,i,j,h,k}) = u\right\}|}\\&\qquad\sum_{\left\{(\alpha,i,j,h,k)|es(T_{\alpha,i,j,h,k}) = u\right\}} x_{\alpha,i,j,h,k}.  \end{array} $$

**Figure 8:**
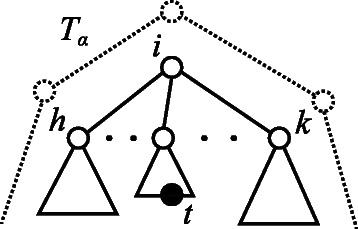
Illustration of subtree *T*_*α*,*i*,*t*,*h*,*k*_ in *T*_*α*_. *T*_*α*,*i*,*t*,*h*,*k*_ denotes the subtree rooted at vertex *v*_*i*_ having the child vertices *v*_*j*_(*h*≤*j*≤*k*) and vertex *v*_*t*_ labeled with a tag in *T*_*α*_.

Here, *l* *c* *h*(*α*,*i*), *r* *c* *h*(*α*,*i*), and *c* *h*(*α*,*i*) denote the leftmost child of the vertex *v*_*i*_ in *T*_*α*_, the rightmost child of *v*_*i*_ in *T*_*α*_, and the set of child vertices of *v*_*i*_ in *T*_*α*_, respectively. *T*_*α*,*i*,*t*,*h*,*k*_ denotes the subtree rooted at vertex *v*_*i*_, with the child vertices *v*_*j*_(*h*≤*j*≤*k*) and *v*_*t*_ labeled with a tag in *T*_*α*_, which does not have a tag when *t*=*ε* (Figure 8). *I*(*T*) denotes the set of internal vertices, except for the root and leaves of tree *T*. *a* *n* *c*(*α*,*j*) denotes the set of ancestor vertices of *v*_*j*_, where *j*∉*a* *n* *c*(*j*) and *a* *n* *c*(*ε*)=*∅*.

In MinSEOTGMul, the variable *p*_*u*_ is equated to 1 if a nonterminal symbol that corresponds to the subtree represented by Euler string *u* appears in the grammar; otherwise, the variable is equated to 0. MinSEOTGMul minimizes the sum of *p*_*u*_, i.e., the number of distinct nonterminal symbols appeared in the output grammar as a result. The variable *x*_*α*,*i*,*t*,*h*,*k*_ takes on a value of 1, if the subtree *T*_*α*,*i*,*t*,*h*,*k*_ is constructed by the grammar; otherwise, the value of this variable remains 0. In Eqs. (1) and (2), *x*_*α*,*i*,*ε*,*j*,*j*_ and *x*_*α*,*i*,*j*,*j*,*j*_ correspond to an edge in *T*_*α*_, and each edge in the *N* trees is always constructed according to a production rule of (RNC). *x*_*α*,1,*ε*,*lch*(*α*,1),*rch*(*α*,1)_ corresponds to the entire *α*-th tree *T*_*α*_, where the root of each tree is numbered as 1. Eq. (3) represents that MinSEOTGMul requires that all *N* trees *T*_*α*_ are constructed using the grammar.

**Figure 9:**
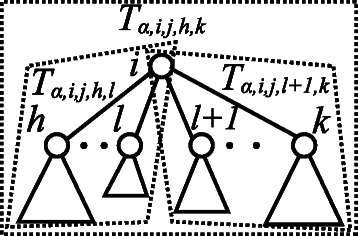
Horizontal bisection of *T*_*α*,*i*,*j*,*h*,*k*_ in *T*_*α*_. The nonterminal symbol corresponding to *T*_*α*,*i*,*j*,*h*,*k*_ is generated if the nonterminal symbols corresponding to subtrees *T*_*α*,*i*,*j*,*h*,*l*_ and *T*_*α*,*i*,*j*,*l*+1,*k*_ are generated.

**Figure 10:**
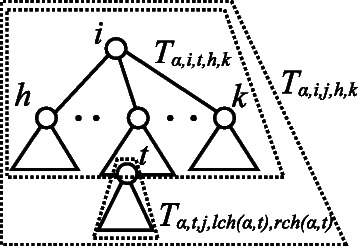
Vertical bisection of *T*_*α*,*i*,*j*,*h*,*k*_ in *T*_*α*_. The nonterminal symbol corresponding to *T*_*α*,*i*,*j*,*h*,*k*_ is generated if the nonterminal symbols corresponding to subtrees *T*_*α*,*i*,*t*,*h*,*k*_ and *T*_*α*,*t*,*j*,*lch*(*α*,*t*),*rch*(*α*,*t*)_ are generated.

The variable *y*_*α*,*i*,*j*,*h*,*l*,*k*_ takes on a value of 1 if *T*_*α*,*i*,*j*,*h*,*k*_ is constructed from *T*_*α*,*i*,*j*,*h*,*l*_ and *T*_*α*,*i*,*j*,*l*+1,*k*_ using an (RHB) production rule; otherwise, the value is maintained at 0 (Figure 9). The variable *z*_*α*,*i*,*j*,*h*,*k*,*t*_ is denoted as 1 if *T*_*α*,*i*,*j*,*h*,*k*_ is constructed from *T*_*α*,*i*,*t*,*h*,*k*_ and *T*_*α*,*t*,*j*,*lch*(*α*,*t*),*rch*(*α*,*t*)_ using an (RVB) production rule; otherwise, the value is retained as 0 (Figure 10). Eqs. (4) and (7) indicate that the subtree *T*_*α*,*i*,*j*,*h*,*k*_ is constructed by at least one established production rule of (RHB) and (RVB) in the grammar. Eqs. (5), (6), (8), (9), and (10) indicate that a production rule of (RHB) and (RVB) becomes a candidate rule in the grammar when both of the two source subtrees are constructed.

The variable *s*_*u*_ is defined by Eq. (12), and takes on a real value of 0≤*s*_*u*_≤1. If at least one subtree *T*_*α*,*i*,*j*,*h*,*k*_ whose Euler string is *u*, i.e., *e* *s*(*T*_*α*,*i*,*j*,*h*,*k*_)=*u*, is constructed, then *s*_*u*_>0. Based on Eq. (11), *p*_*u*_ takes on a value of 1. It means that one nonterminal symbol corresponding to the subtree appears in the grammar. Conversely, when any subtree whose Euler string is *u* is not constructed, then *s*_*u*_=0, *p*_*u*_ takes on a value of 0, and a nonterminal symbol is not generated. In our previous study, unnecessary nonterminal symbols could be generated, and made it difficult to find the minimum number of nonterminal symbols.

For unordered trees, we propose the following IP formulation, MinSEUTGMul, to determine the minimum SEUTG for constructing *N* unordered trees *T*_*α*_, in a manner similar to that used for ordered trees.

$${} {\small{\begin{aligned} &\text{Minimize}\sum_{u\in U}p_{u}\\ &\text{Subject to}\\ &x_{\alpha,i,\epsilon,\{j\}} = 1 \quad \text{ for all } \alpha, i, j\in ch(\alpha,i) (|ch(\alpha,j)|=0),\\ &x_{\alpha,i,j,\{j\}} \,= 1 \quad \text{ for all } \alpha, i, j\in ch(\alpha,i) (|ch(\alpha,j)|>0),\\ &x_{\alpha,1,\epsilon,ch(\alpha,1)}=1 \quad \text{ for all } \alpha,\\ &x_{\alpha,i,\epsilon,{\cal C}}\leq \sum_{{\cal C}'(\neq\emptyset)\subset{\cal C}} y_{\alpha,i,\epsilon,{\cal C}',{\cal C}-{\cal C}'}+\sum_{t\in I(T_{\alpha,i,\epsilon,{\cal C}})}z_{\alpha,i,\epsilon,{\cal C},t}\\ &\hspace*{50pt} \text{for all } \alpha, i, {\cal C}\subseteq ch(\alpha,i), \\ &y_{\alpha,i,\epsilon,{\cal C}',{\cal C}-{\cal C}'}\leq \frac{1}{2}(x_{\alpha,i,\epsilon,{\cal C}'}+x_{\alpha,i,\epsilon,{\cal C}-{\cal C}'}) \\ &\hspace*{50pt}\text{for all } \alpha, i, {\cal C}\subseteq ch(\alpha,i), {\cal C}'(\neq\emptyset)\subset{\cal C},\\ &z_{\alpha,i,\epsilon,{\cal C},t}\leq \frac12(x_{\alpha,i,t,{\cal C}}+x_{\alpha,t,\epsilon,ch(\alpha,t)}) \\ &\hspace*{50pt}\text{for all } \alpha, i, {\cal C}\subseteq ch(\alpha,i), t\in I(T_{\alpha,i,\epsilon,{\cal C}}),\\ &x_{\alpha,i,j,{\cal C}} \leq \sum_{{\cal C}'(\neq\emptyset)\subset{\cal C}} y_{\alpha,i,j,{\cal C}',{\cal C}-{\cal C}'}+\sum_{t\in anc(\alpha, j)}z_{\alpha,i,j,{\cal C},t}\\ &\hspace*{50pt}\text{for all } \alpha, i, {\cal C}\subseteq ch(\alpha,i), j\in I(T_{\alpha,i,\epsilon,{\cal C}}), \\ &y_{\alpha,i,j,{\cal C}',{\cal C}-{\cal C}'}\!\leq\! \frac12(x_{\alpha,i,\epsilon,{\cal C}'}\,+\,x_{\alpha,i,j,{\cal C}-{\cal C}'}) \\ &\hspace*{50pt}\text{for all } \alpha, i, {\cal C}\!\subseteq\! ch(\alpha,i), j\!\in\! I(T_{\alpha,i,\epsilon,{\cal C}}), {\cal C}'(\neq\!\emptyset)\!\subset\!{\cal C},\\ &z_{\alpha,i,j,{\cal C},t}\leq \frac12(x_{\alpha,i,t,{\cal C}}+x_{\alpha,t,j,ch(\alpha,t)}) \\ &\hspace*{50pt}\text{for all } \alpha, i, {\cal C}\!\subseteq\! ch(\alpha,i), j\!\in\! I(T_{\alpha,i,\epsilon,{\cal C}}), t\!\in\! anc(\alpha, j),\\ &s_{u}\leq p_{u}<1+s_{u} \quad \text{ for all } u \in U, \\ &s_{u}= {\frac 1 {|\{(\alpha,i,j,{\cal C})|es(T_{\alpha,i,j,{\cal C}}) = u\}|}} \sum_{\{(\alpha,i,j,{\cal C})|es(T_{\alpha,i,j,{\cal C}}) = u\}} x_{\alpha,i,j,{\cal C}}. \end{aligned}}} $$

The horizontal bisection rules of (RHB) split the set of child vertices of the vertex *v*_*i*_ into a subset ^′^, and the remaining vertices −^′^. *T*_*α*,*i*,*j*,_ indicates that the subtree rooted at vertex *v*_*i*_ has a set of the child vertices. The variables *x*_*α*,*i*,*j*,_, $y_{\alpha,i,j,{\cal C}^{\prime }, {\cal C}-{\cal C}^{\prime }}$, and *z*_*α*,*i*,*j*,,*t*_ are used in a manner similar to *x*_*α*,*i*,*j*,*h*,*k*_, *y*_*α*,*i*,*j*,*h*,*l*,*k*_, and *z*_*α*,*i*,*j*,*h*,*k*,*t*_ in MinSEOTGMul, respectively.

**Figure 11:**
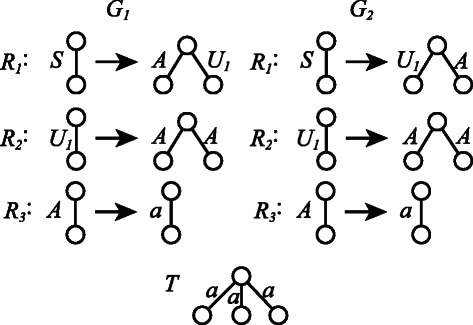
Minimum SEOTGs *G*_1_ and *G*_2_ that generate tree *T*. The number of nonterminal symbols of *G*_1_ (*G*_2_) is 3. The first production rule *R*_1_ of *G*_1_ is different from *R*_1_ of *G*_2_.

It should be noted that the IP formulations MinSEOTGMul and MinSEUTGMul can output multiple grammars with the minimum number of nonterminal symbols. Figure 11 displays such an example, where the grammars *G*_1_ and *G*_2_ generate the tree *T*, and the number of nonterminal symbols of *G*_1_ (*G*_2_) is 3. The first production rule *R*_1_ of *G*_1_ is different from the *R*_1_ of *G*_2_. By providing more such trees, the number of the minimum grammars can be reduced to almost one.

## Results and discussion

### Tree representation of glycans and RNAs

The proposed methods MinSEOTGMul and MinSEUTGMul were evaluated by preparing two types of biological data, glycans and RNA secondary structures, which were dealt with as unordered and ordered trees, respectively. For this analysis, we utilized 16 glycans, G02677, G03655, G03661, G03664, G03678, G03687, G04186, G04458, G04695, G04802, G05058, G05226, G05256, G05988, G07243, and G09054, from the KEGG Glycan database [20]. As glycans are regarded as vertex labeled rooted trees, wherein each vertex is a monosaccharide, the glycans were transformed into edge labeled rooted trees, wherein each edge is labeled with a label of its lower vertex.

**Figure 12:**
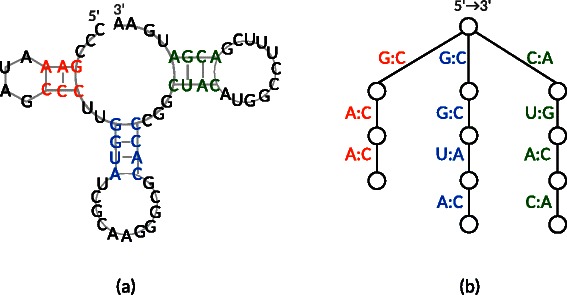
Illustration of RNA tree representation.**(a)** An artificial RNA secondary structure. **(b)** The tree representation of (a) in which paired bases are extracted by following the sequence order from 5’ to 3’, where bases in loops are removed. Base pairs belonging to the same secondary structure are filled in the same color.

In addition, 24 RNA secondary structures belonging to distinct RNA families were taken from the Rfam database [21], as shown in Additional file 1: Table S1 on our supplementary web site; these were transformed into rooted ordered trees. For this, one sequence was selected from multiple sequence alignments of each RNA family, as our method requires edge labels, i.e., bases. RNA secondary structures consist of base pairs with hydrogen bonds, and group binding, such as bulges and hairpin loops (as seen in Figure 12 (a)). There are several representations of trees for RNA secondary structures. An RNA secondary structure can be represented as an ordered rooted tree, by labeling the vertices with unpaired loops and the edges with paired bases [22]; this structure can be represented as an ordered rooted tree by labeling the vertices with hairpin loops, internal loops, bulges, and paired bases [7,23]. Chen and Zhang represented an RNA secondary structure using a paired base and a leaf, corresponding to an internal vertex and an unpaired base, respectively [8]. In our implementation, the representation by [8] was modified by eliminated vertices other than those corresponding to paired bases; in addition, the vertex labeled tree was transformed into an edge labeled tree in a manner similar to the glycans. Figure 12 illustrates the transformation, wherein a paired base was transformed into an edge, labeled with its base pair. It is noted that the edges in this representation are ordered by following the 5’-3’ direction of the RNA sequence.

The CPLEX Optimization Studio (version 12.5) was used to solve integer programming using a linux operating system. The source code to transform given multiple trees into the proposed IP formulations is available at our supplementary web site on http://sunflower.kuicr.kyoto-u.ac.jp/tyoyo/treecomp/.

### Minimum grammars for glycans and RNAs

**Figure 13:**
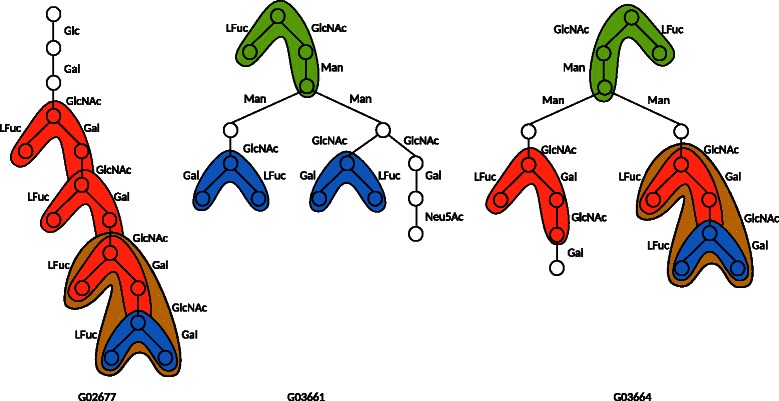
Results of subtrees corresponding to nonterminal symbols in the minimum SEUTG for unordered rooted trees of glycans G02677, G03661, G03664. Subtrees corresponding to the same nonterminal symbol are filled in the same color. ’Glc’, ’Gal’, ’GlcNAc’, ’LFuc’, ’Man’, and ’Neu5Ac’ denote glucose, galactose, N-acetylglucosamine, L-fucose, mannose, and N-acetylneuraminic, respectively.

We applied the proposed method MinSEUTGMul for the glycans and their several combinations. Table 1 shows the minimum number of nonterminal symbols of SEUTG for glycan unordered trees. In all cases, the minimum number of nonterminal symbols for multiple trees of glycans was lower than the sum of the minimum numbers for its single trees. For example, the minimum number of nonterminal symbols for G02677, G03661, and G03664 was 31, which was lower than the sum of the minimum numbers 13+17+16=46. This suggests that our method successfully determined several common rules among the combination of glycans, and that the compression of several glycans together is better than that of each individual glycan. The execution time and the memory usage by the IP solver, for multiple trees with over 100 vertices in our experiments, were observed to be less than 10 minutes and 4G bytes, respectively (except in the case of G02677, G03661, G04458, and G07243). These compression sizes can be used to estimate the similarities between glycan structures. If the compression size of two glycans is smaller than the sum of compression sizes of the individual glycans, these glycan structures are considered to be similar. Figure 13 shows the subtrees corresponding to the nonterminal symbols contained within the minimum SEUTG for the unordered rooted trees of the glycans G02677, G03661, and G03664; the subtrees corresponding to the same nonterminal symbol are filled with the same color, and a portion of the nonterminal symbol is shown. The nonterminal symbol colored blue appeared in all three glycans, while those colored green and red appeared in two glycans. The nonterminal symbol colored brown consisted of the nonterminal symbols colored red and blue. This implies that the hierarchical structures contained within the glycans beyond the frequent patterns can be extracted using the developed methods.

**Table 1:** **Results on the minimum number of nonterminal symbols by MinSEUTGMul for glycan unordered trees**

**Glycan**	**#vertices**	**Sum**	**Min**	**Time (sec)**	**Memory (MB)**
G02677	15		13	4.2	173.75
G03655	34		26	35.94	755.59
G03661	15		17	1.76	171.56
G03664	17		16	4.17	178.46
G03678	18		20	2.33	212.38
G03687	28		17	77.97	930.05
G04186	20		19	2.64	216.28
G04458	21		9	3.96	207.81
G04695	20		17	2.31	196.89
G04802	20		15	5.39	189.74
G05058	25		16	5.65	221.36
G05226	19		14	5.24	173.63
G05256	25		11	4.62	222.64
G05988	19		18	5.27	204.9
G07243	18		15	4.58	171.89
G09054	31		17	5.06	290.62
G02677, G03661, G03664	47	46	31	268.06	948.56
G03661, G03678, G04186	53	56	44	26.97	681.83
G03678, G03687, G04186	66	56	52	66.45	791.04
G04695, G05058, G05226	64	47	44	137.87	1080.9
G04458, G04695, G04802	61	41	28	408.87	3088.68
G05988, G07243, G09054	68	50	43	68.29	942.43
G02677, G03661, G03664, G03678	65	66	47	96.14	923.62
G02677, G03661, G04458, G07243	69	54	39	8748	16814.68
G03661, G03678, G04186, G05226	72	70	49	56.62	1068.76
G04458, G04695, G04802, G05226	80	55	40	97.1	954.66
G02677, G03661, G03664, G03678, G07243	83	81	52	181.19	955.31
G03661, G03678, G04186, G05226, G05988	91	88	51	51.52	804.57
G03664, G04802, G05256, G05988, G07243	99	75	49	425.89	1717.89
G04458, G04695, G04802, G05226, G05988	99	73	41	164.41	1119.74
G02677, G03661, G03664, G03678, G05226, G07243	102	95	53	220.73	976.34
G03661, G03678, G04186, G05226, G05988, G07243	109	103	53	50.42	1018.3

‘#vertices’ denotes the total number of vertices in glycan trees. ‘sum’ denotes the sum of the minimum numbers for single glycans in a combination, and is omitted for single glycans. ‘min’ denotes the minimum number for all glycans in a combination. ‘time’ denotes the execution time in seconds. ‘memory’ denotes the memory usage in mega bytes.

**Figure 14:**
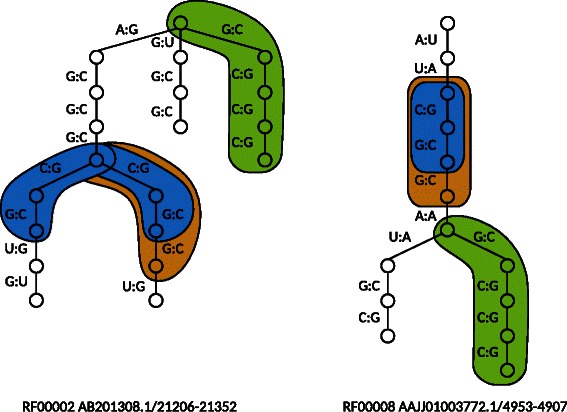
Results of subtrees corresponding to nonterminal symbols in the minimum SEOTG for ordered rooted trees of RNAs from RF00002 and RF00008. Subtrees corresponding to the same nonterminal symbol are filled in the same color.

**Figure 15:**
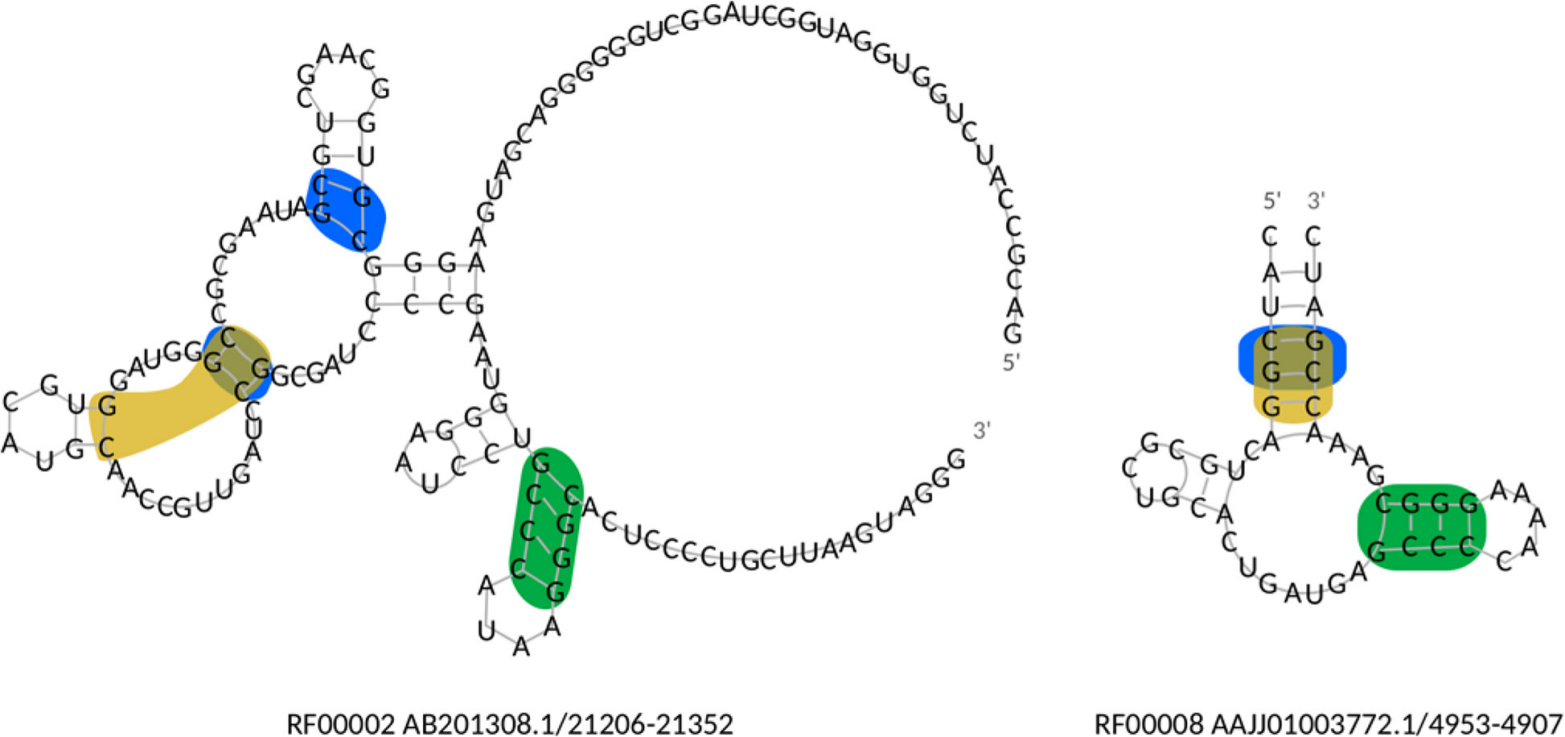
Original RNA secondary structures of RF00002 and RF00008, and base pairs corresponding to nonterminal symbols in the minimum SEOTG. Base pairs corresponding to the same nonterminal symbol are filled in the same color as Figure 14.

The proposed method MinSEOTGMul was applied to the RNA secondary structures and their several combinations. Table 2 shows the minimum number of nonterminal symbols of SEOTG for RNA ordered trees. In all cases, the minimum number of nonterminal symbols for multiple trees of RNA was lower than the sum of minimum numbers for its single trees, similar to the glycans. The execution time and the memory usage by the IP solver for multiple trees in our experiments were between two seconds and six hours, and between 260 Mbytes and 37 Gbytes, respectively. Figure 14 shows the subtrees corresponding to nonterminal symbols in the minimum SEOTG for ordered rooted trees of the RNA secondary structures RF00002 and RF00008. Figure 15 shows the original RNA secondary structures of RF00002 and RF00008, and the base pairs corresponding to nonterminal symbols in the minimum SEOTG. We also observed the hierarchical structure of the nonterminal symbols, colored blue and brown.

**Table 2:** **Results on the minimum number of nonterminal symbols by MinSEOTGMul for RNA ordered trees**

**RNA**	**#vertices**	**Sum**	**Min**	**Time (sec)**	**Memory (MB)**
RF00002	20		26	57.77	593.89
RF00003	23		31	4836	8186.96
RF00004	14		18	0.5	194.7
RF00005	22		24	411.9	957.51
RF00007	16		21	4.27	261.98
RF00008	14		18	7.51	212.37
RF00016	8		11	0.13	22.49
RF00029	20		26	1010.53	1592.98
RF00032	7		9	0.06	22.18
RF00050	24		25	1993.69	1721.94
RF00072	15		19	8.04	579.91
RF00101	18		22	44.38	562.54
RF00137	8		11	0.09	22.56
RF00166	11		13	0.09	23.3
RF00167	21		24	2099.08	2025.98
RF00234	21		24	531.7	920.3
RF00360	7		9	0.05	22.24
RF00442	18		21	23.99	585.23
RF00517	13		14	0.58	170.37
RF00519	13		10	0.28	25.9
RF01054	18		19	9.47	502.48
RF01829	9		11	0.08	22.47
RF01850	20		21	68.63	710.26
RF01851	21		23	460.43	1066.23
RF00002, RF00004	34	44	37	4329	3962.39
RF00002, RF00008	34	44	36	1861.88	2702.14
RF00003, RF00004	37	49	43	5600	9553.93
RF00004, RF00007	30	39	34	2104.57	3023.84
RF00004, RF00016	22	29	23	2.04	264.36
RF00005, RF00016	30	35	30	61.19	5326.1
RF00007, RF00008	30	39	34	922.44	4152.85
RF00007, RF00032	23	30	26	2.46	282.71
RF00016, RF00029	28	37	31	106.51	13453.93
RF00016, RF00072	23	30	21	5.76	486.06
RF00029, RF00360	27	35	31	142.6	3537.38
RF00032, RF00050	31	34	30	3405.65	5729.7
RF00050, RF00137	32	36	35	214.27	3325.72
RF00050, RF01829	33	36	29	7958	11276
RF00072, RF00137	23	30	28	6.2	527.51
RF00072, RF01829	24	30	27	6.49	542.42
RF00101, RF01829	27	33	29	244.58	1004.71
RF00166, RF00167	32	37	32	19138	36018.49
RF00166, RF01054	29	32	28	151.13	851.54
RF00360, RF00442	25	30	27	47.75	593.08
RF00442, RF00519	31	31	26	81.07	759.43
RF00517, RF00519	26	24	22	3.38	403.29
RF01850, RF01851	41	44	23	220.6	1290.55
RF00002, RF00008, RF00032	41	53	40	2209.81	4734.47
RF00002, RF00004, RF00016	42	55	41	10189	17355.98
RF00004, RF00007, RF00016	38	50	38	4605	8973.44
RF00004, RF00008, RF00032	35	45	32	135.14	733.37
RF00007, RF00008, RF00032	37	48	38	821.59	2310.18
RF00007, RF00016, RF00032	31	41	33	66.79	646.84
RF00016, RF00072, RF00137	31	41	30	4.56	353.48
RF00032, RF00137, RF00234	36	44	39	601.1	2105.12
RF00137, RF01850, RF01851	49	55	32	239.55	1472.98
RF00517, RF00519, RF01054	44	43	35	3020.41	6465.58
RF00004, RF00016, RF00072, RF00137	45	59	41	976.24	2862.5

’#vertices’ denotes the total number of vertices in trees transformed from RNAs. ’sum’ denotes the sum of the minimum numbers for single RNAs in a combination, and is omitted for single RNAs. ’min’ denotes the minimum number for all RNAs in a combination. ’time’ denotes the execution time in seconds. ’memory’ denotes the memory usage in mega bytes.

We examined the alternative approach that transforms multiple trees into a single tree and applies our previous methods. For the set of glycans G02677, G03661, and G03664, the alternative method output the existence of the SEUTG grammar with size 39 in 8.83 seconds. However, we could not obtain the result for size 38 within 24 hours, and could not determine the minimum number of nonterminal symbols. For the set of RNAs RF00002, and RF00004, the method output the existence of the SEOTG grammar with size 42 in 5.01 seconds. However, we could not obtain the result for size 41. We can see that the proposed methods are more efficient than the previous methods.

## Conclusions

We proposed novel integer programming-based methods MinSEOTGMul and MinSEUTGMul to determine the minimum simple elementary ordered and unordered tree grammars (SEOTG and SEUTG) for multiple ordered and unordered trees, respectively. These could be directly applied to the determination of the minimum grammar, unlike our previously proposed methods. We applied MinSEUTGMul to several unordered trees transformed from glycans, and their combinations; MinSEOTGMul was applied to several ordered trees transformed from RNA secondary structures, and their combinations. In all cases, the minimum number of nonterminal symbols in the grammars used in the construction of multiple trees was lower than the sum of minimum numbers in the grammars used to construct the single trees. This suggests that the proposed methods were successful in determining several common rules for glycans and RNA. In addition, several results of the minimum grammars for multiple trees of glycans and RNA reveal that our methods can provide clues towards extracting the hierarchical structures contained within tree-structured biological data, beyond the frequent patterns.

In our experiments, the execution time and the memory usage for a set of trees required six hours and 37GBytes, respectively. To obtain the minimum SEOTG and SEUTG for more trees including more complicated trees, we need to further improve the efficiency.

In this study, we utilized the minimum grammar for extraction of common construction rules among multiple distinct trees. However, the proposed methods can be used for data compression. Furthermore, the execution times of some operations can be decreased to multiple trees by applying the operations to the previously obtained minimum grammar. In the future, we would like to apply our methods to more glycans, RNA, and other tree-structured biological data.

## Additional file

Additional file 1
**Table S1.** RNA sequences used in our experiments.
