# Supplementary material for: Grammar-based compression approach to extraction of common rules among multiple trees of glycans and RNAs
Source: BMC Bioinformatics. 2015 Apr 24;16:128. doi: 10.1186/s12859-015-0558-4 (PMC4419412; doi:10.1186/s12859-015-0558-4)
Supplement: Additional file 1 — Table S1. RNA sequences used in our experiments. [file 12859_2015_558_MOESM1_ESM.pdf]

RNA family	ID	Selected RNA	Sequence
RF00002	5_8S_rRNA	AB201308.1/ 21206-21352	GACGCCAUCUGGUGGAUGGCUAGGCUGGGGGGACGAUGAAGGGCGUGGCAAGCUGCGAUAAGCGCCGGGUAGGUGCAUGCAACCGUUGAUCCGGCGAUCCCCGAAUGGGAAUCCUGCCCAUAAGGGCACUCCCCCUGCUUAAGUAGGG
RF00003	U1	ADND01229876.1/ 179-302	AUACUUAUCUGGCUAGUAGGUAAACCGUGAUCAAUAAGGCGGUUCAUCAGGGGCAAGGGCCUUCCAUUACACUUAGUUGGGCUUAAUGCGGAUAACACUAAACAAACAAAUUUCUGGUGAUUUC
RF00004	U2	AFXY01000623.1/ 84-1	AUACCUUCUCGGCCUUUUGGCUAAGAUCAAGUGUAGUAUCUGUUCUUAUCAGUGUGAAAACUGAUACUGUCCCUACUAGGGACA
RF00005	tRNA	EU826467.1/ 91340-91413	AGCGGUGUAGAGCAGCUAGGUAGCUCGCCGGGCUCAUGACCCGGAGGACGCGUGUUCGAUUCACGCCACCGCCA
RF00007	U12	ACPB02042234.1/ 26674-26749	GCACCUUAAACUAAUGAGUAAGGAAAAUAUUGAGCCUUGUUAACUUUGGGUUCAGCUACCAACUUUAAGUAUAUCC
RF00008	Hammerhead_3	AAJJ01003772.1/ 4953-4907	CAUCGGACUGCGCUGCACUGAUGAGCCCCAAAAGGGCGAAACCGAUC
RF00016	SNORD14	AAEL01000370.1/ 5957-6071	UUUCAAUGAUGACAAAAAUUAACGUUUCUCAACAUUCGCAGUGUUGUACCGAAAUGGAGAUCUUAAAUAUGCGCAUUUAAGACUCUAUGAUGGACUUCCUUAGAUGUCUGAGAGA
RF00029	Intron_gpII	CP000300.1/ 803648-803724	UUGAGCUGUGUGAGGUGAAAGUCUCACGCACGGUUCUUAGGCGAGAAAGAGGGAGUAAUCUCUCUGACUUAGCCGAC
RF00032	Histone3	AAJT02000277.1/ 837-792	AAAUCGGUCCUUUUCAGGACCACUAAAGUUAUUAAUCAAAAGAGAA
RF00050	FMN	AELY01000021.1/ 146236-146106	UACAAUUCUCAGGGCGGAGUGAAAUUCUCCACCGGUGGUGUUUCUAAAUAGAUAAGUCCACGAGCACAAAAUGUGUUGAUUUGGUUAGAGUCCAAAACCGACGGUUAUAGUCCGGAUGGAAGAGAAUAAGA
RF00072	SNORA75	CAEC01338178.1/ 1038-1118	CACUAGAAGACAGAAUUCACAGAAGUAGCAUUUCACCUUUUGUCUUUACAGAAGUAUAUUUGGCUGUUUUGUGAGACAUUC
RF00101	SraC_RyeA	AGFT01130371.1/ 55-1	GACCGAAUACGAUUCCUGUAUUCGGUCCAGGGAAAUGGCUCUUGGGAGAGAGCCG
RF00137	SNORD83	CR792441.21/ 87264-87189	GCCACAUGAUGUGAUGUAGAUUGCAUUAGCAUUGCUGCAAAGAUGACCGUUUAUCUAUCACCCAUGACUGAUGGCU
RF00166	PrrB_RsmZ	HQ395566.1/91-56	AAGGACAUCGCAGGACGCGAUUCAUCAGGAUGAUGA
RF00167	Purine	CP002902.1/ 521634-521700	CUCGUAUAAUGCCGGGAAUAUGGCCCGGCAGUCUCUACGAGGCGACCGUAAAUCGCCUUGCUACGAG
RF00234	glmS	ADXE01000882.1/ 23-92	CGGCUGUGCAUGCAGUCGUUAAGUCUUACUUACAAAUCACUUGGGUGACCAAGUGGACAGAGUAGUAAUG
RF00360	snoZ107_R87	AENI01000318.1/ 969-858	GAAGGCAGUGACGACUCGGAAAUAUUCAAGCUCAACAAACCGGAACUAGGCGUUUCUUCAUUUAACUUGGAGAUUCGUUUAUGUGUCGAUAAUCCCGCUGAACUGAGCCUUU
RF00442	ykkC-yxkD	AEGE01001314.1/ 179-278	GUGGGGCGCGUCUAGGGUUCCGUCCCCUUGCGGGAGUCUGGUCCGAGAGACGCAGACGGCUUGACGAGCCGUCCCACGGCGGGACAAAAGCCCGGGAGAC
RF00517	serC	BX572606.1/ 323831-323780	CAGCGGGAAACCUCUCCCCACCGAGAGGCAGCUAUCUGGAAGGAUAGACUAU
RF00519	suhB	CP002102.1/ 747290-747214	GCGUCUUGCCCUUCCUGGGCGUUUCCUCCCUAUAGACUUUAUGCCGCGCCUUCGGGCGCGGCUUUUUUUCGUCUGGU
RF01054	preQ1-II	AGHQ01000118.1/ 6352-6451	UAUGCUAUACUAGAACAGCUGGUUUCCAGCCCUUGGUGCUUAGCUCUUUUCACCAAGCAUAUUUUAAGCGGUCGCCCCGCUAAAGGAGAAUCAUCACAUG
RF01829	sR6	CP000077.1/ 368187-368239	GGAUGAUGACCAAAUAGACUGAAAGAUGAAGAAAUGCACCUCAACUGACUAAA
RF01850	beta_tmRNA	AY082655.1/ 3-105	UGUCUCGGACAGCGGUUCGAUUCCGCUCAGCUCCAUUCCCCGACCCACCGCCCCGCCGGCGGUGCGUCAUCCCGGGGCUGCAAUGGUUUCGACGGGGCAUGA
RF01851	cyano_tmRNA	AY082655.1/ 1-105	AGUGUCUCGGACAGCGGUUCGAUUCCGCUCAGCUCCAUUCCCCCGACCCACCGCCCCGCCGGCGGUGCGUCAUCCCGGGGCUGCAAUGGUUUCGACGGGGCAUGA
